# Analysis of Delayed Surgical Treatment and Oncologic Outcomes in Clinical Stage I Non–Small Cell Lung Cancer

**DOI:** 10.1001/jamanetworkopen.2021.11613

**Published:** 2021-05-27

**Authors:** Brendan T. Heiden, Daniel B. Eaton, Kathryn E. Engelhardt, Su-Hsin Chang, Yan Yan, Mayank R. Patel, Daniel Kreisel, Ruben G. Nava, Bryan F. Meyers, Benjamin D. Kozower, Varun Puri

**Affiliations:** 1 Division of Cardiothoracic Surgery, Department of Surgery, Washington University School of Medicine in St Louis, Missouri; 2 VA St Louis Health Care System, St Louis, Missouri; 3 Division of Public Health Sciences, Department of Surgery, Washington University School of Medicine in St Louis, Missouri

## Abstract

**Importance:**

The association between delayed surgical treatment and oncologic outcomes in patients with non–small cell lung cancer (NSCLC) is poorly understood given that prior studies have used imprecise definitions for the date of cancer diagnosis.

**Objective:**

To use a uniform method to quantify surgical treatment delay and to examine its association with several oncologic outcomes.

**Design, Setting, and Participants:**

This retrospective cohort study was conducted using a novel data set from the Veterans Health Administration (VHA) system. Included patients had clinical stage I NSCLC and were undergoing resection from 2006 to 2016 within the VHA system. Time to surgical treatment (TTS) was defined as the time between preoperative diagnostic computed tomography imaging and surgical treatment. We evaluated the association between TTS and several delay-associated outcomes using restricted cubic spline functions. Data analyses were performed in November 2021.

**Exposure:**

Wait time between cancer diagnosis and surgical treatment (ie, TTS).

**Main Outcomes and Measures:**

Several delay-associated oncologic outcomes, including pathologic upstaging, resection with positive margins, and recurrence, were assessed. We also assessed overall survival.

**Results:**

Among 9904 patients who underwent surgical treatment for clinical stage I NSCLC, 9539 (96.3%) were men, 4972 individuals (50.5%) were currently smoking, and the mean (SD) age was 67.7 (7.9) years. The mean (SD) TTS was 70.1 (38.6) days. TTS was not associated with increased risk of pathologic upstaging or positive margins. Recurrence was detected in 4158 patients (42.0%) with median (interquartile range) follow-up of 6.15 (2.51-11.51) years. Factors associated with increased risk of recurrence included younger age (hazard ratio [HR] for every 1-year increase in age, 0.992; 95% CI, 0.987-0.997; *P* = .003), higher Charlson Comorbidity Index score (HR for every 1-unit increase in composite score, 1.055; 95% CI, 1.037-1.073; *P* < .001), segmentectomy (HR vs lobectomy, 1.352; 95% CI, 1.179-1.551; *P* < .001) or wedge resection (HR vs lobectomy, 1.282; 95% CI, 1.179-1.394; *P* < .001), larger tumor size (eg, 31-40 mm vs <10 mm; HR, 1.209; 95% CI, 1.051-1.390; *P* = .008), higher tumor grade (eg, II vs I; HR, 1.210; 95% CI, 1.085-1.349; *P* < .001), lower number of lymph nodes examined (eg, ≥10 vs <10; HR, 0.866; 95% CI, 0.803-0.933; *P* < .001), higher pathologic stage (III vs I; HR, 1.571; 95% CI, 1.351-1.837; *P* < .001), and longer TTS, with increasing risk after 12 weeks. For each week of surgical delay beyond 12 weeks, the hazard for recurrence increased by 0.4% (HR, 1.004; 95% CI, 1.001-1.006; *P* = .002). Factors associated with delayed surgical treatment included African American race (odds ratio [OR] vs White race, 1.267; 95% CI, 1.112-1.444; *P* < .001), higher area deprivation index [ADI] score (OR for every 1 unit increase in ADI score, 1.005; 95% CI, 1.002-1.007; *P* = .002), lower hospital case load (OR for every 1-unit increase in case load, 0.998; 95% CI, 0.998-0.999; *P* = .001), and year of diagnosis, with less recent procedures more likely to have delay (OR for each additional year, 0.900; 95% CI, 0.884-0.915; *P* < .001). Patients with surgical treatment within 12 weeks of diagnosis had significantly better overall survival than those with procedures delayed more than 12 weeks (HR, 1.132; 95% CI, 1.064-1.204; *P* < .001).

**Conclusions and Relevance:**

Using a more precise definition for TTS, this study found that surgical procedures delayed more than 12 weeks were associated with increased risk of recurrence and worse survival. These findings suggest that patients with clinical stage I NSCLC should undergo expeditious treatment within that time frame.

## Key Points

**Question:**

What is the association between delayed surgical treatment and oncologic outcomes among patients with non–small cell lung cancer (NSCLC)?

**Findings:**

In this retrospective cohort study of 9904 patients with clinical stage I NSCLC using data from the Veterans Health Administration, surgical procedures that were delayed more than 12 weeks from the date of radiographic diagnosis were associated with increased risk of recurrence and worse overall survival.

**Meaning:**

These findings suggest that patients with clinical stage I NSCLC should receive surgical treatment within at least 12 weeks of radiographic diagnosis.

## Teaser

This cohort study examines the association between delayed surgical treatment and recurrence and survival among patients with non–small cell lung cancer in a cohort of patients treated in the Veterans Health Administration system.

## Introduction

Surgical resection remains the standard treatment for early-stage non–small cell lung cancer (NSCLC) management.^1^ Patients with NSCLC should receive timely treatment given that delayed surgical treatment holds a theoretical risk for disease progression and therefore poor prognosis.^2,3^ While several studies^4,5,6,7^ found that delayed surgical treatment is associated with worse outcomes, other studies^8,9,10^ found no association. Therefore, whether there is a safe period of time between lung cancer diagnosis and surgical treatment, or treatment in general, remains unclear.

The issue of delayed surgical treatment was further exacerbated in March 2020 when the World Health Organization declared COVID-19 to be a global pandemic.^11^ In line with several local and state mandates, the American College of Surgeons, Society of Thoracic Surgery, and American Association for Thoracic Surgery authored a consensus statement for delaying surgical treatment during the pandemic.^12^ To support that policy, which generally recommended proceeding with lung cancer resections, a prior study^4^ from our group was cited in which patients with clinical stage I NSCLC in the National Cancer Database (NCDB) were found to have a higher likelihood of upstaging and worse overall survival when surgical procedures were delayed more than 8 weeks from the time of diagnosis. Several additional studies^6,7^ in the NCDB found similar associations, although the definition of unsafe delay ranged from 5 to 13 weeks, depending on what (often arbitrary) time cutoff studies used to define delayed surgical treatment. This wide range of estimates makes counseling patients difficult even in nonpandemic periods. Delayed surgical treatment imposes a particularly stressful burden at academic and Veterans Health Administration (VHA) medical centers, where patients are known to wait longer for operations, with unclear consequences.^13,14^

While several studies have used the NCDB to analyze delayed surgical treatment, the database is not without its own flaws. Time to surgical treatment (TTS) is calculated based on the time between the date of cancer diagnosis and the date of surgical treatment. Coders in the NCDB (and other large national databases, such as the VHA database, which share common coding rules) code this date of diagnosis based on imprecise “clinical” or “microscopic” criteria as determined by a “medical practitioner.”^15^ The ambiguity of this definition allows for a wide range of actually coded dates based on radiologic, clinical, or histologic criteria, which can even include the day of surgical treatment. The imprecise nature of how this date was determined for each patient makes conclusions about delayed surgical treatment from this database uncertain and prone to misclassification bias.

We performed a retrospective cohort analysis of patients with clinical stage I NSCLC receiving surgical treatment in the VHA. The objectives of this study were to adopt a more precise method to quantify TTS based on a universal radiological definition and to examine the association between TTS and several delay-associated and cancer-specific outcomes, including pathologic upstaging, resection with positive margins, and recurrence. We hypothesized that longer TTS would be associated with adverse cancer-associated outcomes, including pathologic upstaging and recurrence.

## Methods

This retrospective cohort study protocol was reviewed and approved by the St. Louis VHA Research and Development Committee and was deemed exempt from institutional review board approval and informed consent given the deidentified nature of the analysis. Data were reported according to the Strengthening the Reporting of Observational Studies in Epidemiology (STROBE) reporting guideline.

The study was performed using the US Veterans Administration (VA) Informatics and Computing Infrastructure system, which contains clinical and administrative data from multiple platforms within the VA's Corporate Data Warehouse (CDW). Adult patients with clinical stage I NSCLC undergoing resection from October 2006 through September 2016 were eligible. All patients underwent surgical treatment and were required to undergo preoperative computed tomography (CT) imaging. Exclusion criteria were large tumor size (ie, >5 cm [>T1-2]), receipt of neoadjuvant chemotherapy, extremely delayed surgical treatment (more than 6 months between diagnosis and surgical procedure), and receipt of an operation for recurrent disease.

The time between the date of diagnosis and the date of surgical procedure, or TTS, served as the primary independent variable of interest in this study. This variable was defined in 2 ways based on how the date of diagnosis was defined (ie, radiologically or clinically). First, radiological TTS (RTTS) was defined based on the most recent CT scan before surgical treatment. Scans were identified based on *International Classification of Diseases, Ninth Revision *(*ICD-9*) or *Tenth Revision *(*ICD-10*) procedure or Current Procedural Terminology codes. We preferentially evaluated scans that were performed within 30 to 180 days prior to surgical treatment. Within that time, the scan that occurred closest to the date of surgical treatment was chosen as the date of diagnosis. For patients with CT scans within only 30 days of surgical treatment, that scan was chosen as the date of diagnosis. Second, clinical TTS (CTTS) was defined based on the date of initial cancer diagnosis originally coded in the CDW system (ie, “whether clinically or histologically established” using the same coding standard as the NCDB^16^). We recorded RTTS and CTTS in days. Owing to the inherently imprecise and subjective nature of coding associated with CTTS, we used RTTS in the primary analysis. However, we performed a sensitivity analysis using the CTTS variable as well.

Patient characteristics were extracted from the VHA system; these included age, sex, body mass index (BMI; calculated as weight in kilograms divided by height in meters squared), race (as coded in the CDW, defined according to the American College of Surgery Facility Oncology Registry Data Standards), area deprivation index (ADI) score, median household income, median high school graduation rate at the county level, smoking status (measured 2 weeks before operation), distance from care facility, and comorbidities. Race was included to assess potential factors associated with delayed surgical treatment. The ADI variable is a geography-based measure of socioeconomic deprivation based on 17 US Census poverty, education, housing, and employment indicators that has previously been associated with worse lung cancer outcomes.^17,18^ Comorbidity burden was measured using the Charlson-Deyo Comorbidity Index (CCI) based on *ICD-9* and *ICD-10* codes within 5 years of the date of surgical treatment.^19,20^ We also abstracted operation-specific and tumor-specific characteristics, including tumor size, tumor histology, tumor grade, hospital volume (measured as number of patients with a lung cancer diagnosis at that institution in the year prior to the patient’s surgical procedure), year of operation, performance of invasive mediastinal staging (ie, endobronchial ultrasound or mediastinoscopy), incision type (ie, video-assisted thoracoscopic surgical procedure or thoracotomy), operation type (ie, lobectomy, segmentectomy, wedge resection, or pneumonectomy), and number of lymph nodes examined.

The primary outcomes were pathologic upstaging (ie, stage II-IV disease on final pathology report), positive surgical margins (ie, microscopic, macroscopic, or not otherwise specified), and cancer recurrence. Recurrence was defined and verified by several mechanisms in line with prior VHA literature.^21^ First, documented episodes of recurrence were abstracted from VHA records. Second, patients with any of the following occurrences based on *ICD* codes were also deemed to have recurred: additional chemotherapy, additional radiation therapy, additional lung resection, malignant pleural effusion, a secondary malignant neoplasm from lung cancer, or biopsy showing recurrent lung cancer. For patients with pathologic stage I cancer, this follow-up for recurrence started immediately after surgical treatment. For patients with pathologic stage II-III disease, this follow-up for recurrence started 6 months after surgical treatment (and at least 60 days following the last adjuvant chemotherapy or radiation therapy). Patients with pathologic stage IV cancer were not included in the recurrence analysis.

### Statistical Analysis

For upstaging and positive margins, multivariable logistic regression models using restricted cubic spline functions were used to examine the association of TTS with each of these outcomes, controlling for age, race, sex, ADI score, smoking status, CCI score, performance of invasive mediastinal staging, type of operation, incision type, tumor size, tumor grade, number of lymph nodes examined, histology, year of operation, and institutional volume. Multivariable competing-risk models using restricted cubic spline functions were used to examine the association of TTS with risk of recurrence, controlling for readmissions and final pathologic stage (excluding patients with stage IV cancer) in addition to covariates included in the previous models. Knot locations are available in the eAppendix in the Supplement. This restricted cubic spline method holds many advantages in that it makes no assumption regarding the shape of the function.^22,23^ Splines generate nonlinear models between a continuous independent variable (like TTS) and a dependent outcome (like recurrence) without the requirement of categorizing the independent variable.^7,24^ The shapes of the curves were then used to determine if there was an inflection point at which time the risk of worse outcomes significantly increased.

Additional multivariable logistic regression analysis was performed to assess factors associated with delayed surgical treatment. Overall survival was assessed using the Kaplan-Meier method. Observations with missing values were excluded (except tumor size, lymph node collection, and incision when an unknown variable was created). Baseline cohort descriptive statistics were expressed as mean (SD) or absolute number (percent). For nonnormally distributed continuous variables, median with interquartile range (IQR) was reported. *P* values of less than .05 were considered statistically significant, and *P* values were 2-tailed. All data analyses were performed using SAS statistical software version 9.3 (SAS Institute) in November 2021.

## Results

**Figure 1.**
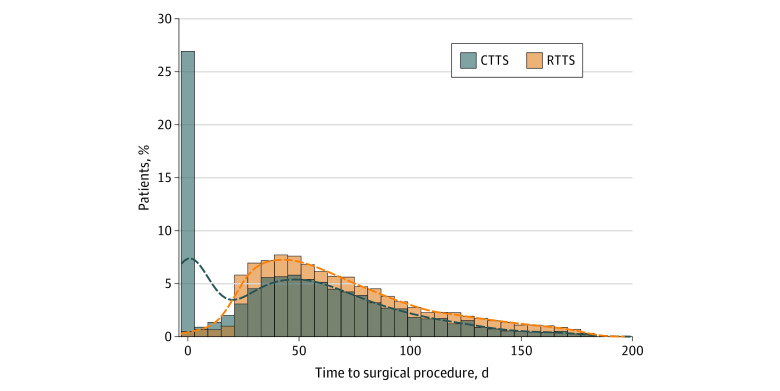
Quantifying Delayed Surgical Treatment by Clinical or Radiological Methods This histogram presents RTTS (radiological time to surgical treatment; based on time between the date of diagnostic computed tomography imaging and surgical treatment) and CTTS (clinical time to surgical treatment; based on time between the date of diagnosis as previously coded in the Veterans Health Administration system and surgical treatment) for patients with clinical stage I non–small cell lung cancer.

Among 9904 patients who met inclusion criteria (Table 1), the mean (SD) age was 67.7 (7.9) years, 9539 (96.3%) were men, and 4972 individuals (50.5%) were currently smoking at the time of surgical treatment. The mean (SD) RTTS was 70.1 (38.6) days; by comparison, the mean (SD) CTTS was 48.5 (41.9) days. According to the traditional CTTS definition, 2453 patients (26.6%) had a 0-day wait time between diagnosis and surgical treatment, suggesting that a large proportion of these patients were diagnosed with cancer on the same day as their operation (Figure 1), a finding consistent with data from the NCDB.^7^ Because these findings may denote patients undergoing surgical treatment without a definitive preoperative histologic diagnosis or with potential coding errors, we chose to continue our analysis using RTTS, which consistently and reliably provided a uniformly applicable diagnostic date (Figure 1).

**Table 1.** Demographic Characteristics of Veterans Affairs Patients, 2006-2016

Characteristic	Patients, No. (%) (N = 9904)
Age, mean (SD), y	67.71 (7.93)
Sex	
Men	9539 (96.31)
Women	365 (3.69)
BMI, mean (SD)	27.18 (5.41)
White race	8225 (83.82)
Area Deprivation Index score, median (IQR)	55.56 (43.03-65.04)
County household income, median (IQR), $	47 628 (41 585-55 452)
County high school graduation rate	78.39 (9.27)
Charlson Comorbidity Index score, median (IQR)	7 (5-8)
Smoking status	
Former	4800 (48.75)
Current	4972 (50.49)
Perioperative characteristic	
Yearly hospital case load, median (IQR)	106 (74-160)
Endobronchial ultrasonography or mediastinoscopy	1739 (17.56)
Wait time from CT scan to surgical procedure (RTTS), d	
Mean (SD)	70.08 (38.58)
Median (IQR)	62 (40-92)
Tumor size, mean (SD), mm	27.32 (10.64)
Resection	
Lobectomy	6923 (70.10)
Wedge	2172 (21.99)
Segment	622 (6.30)
Pneumonectomy	159 (1.61)
Incision type	
Video-assisted thoracoscopic surgical procedure	4670 (53.27)
Thoracotomy	4096 (46.73)
Lymph node collection	
<10	6215 (68.99)
≥10	3045 (31.01)
Histology	
Adenocarcinoma	5236 (52.88)
Squamous	3366 (34.00)
Other	1299 (13.12)
Grade	
I	1195 (13.05)
II	4808 (52.49)
III	3022 (32.99)
IV	135 (1.47)
Outcomes	
30-d mortality	203 (2.05)
30-d readmission	810 (8.18)
Major complications	
Pneumonia	618 (6.24)
Empyema	87 (0.88)
Respiratory or cardiac failure	534 (5.39)
Myocardial infarction	97 (0.98)
Pulmonary embolism	53 (0.54)
Prolonged ventilation (>48 h)	387 (3.91)
Pathologic upstaging	1171 (12.33)
Positive surgical margins	309 (3.12)
Recurrence	4158 (41.98)

Abbreviations: BMI, body mass index (calculated as weight in kilograms divided by height in meters squared); CT, computed tomography; IQR, interquartile range; RTTS, radiological time to surgical treatment.

Most veterans underwent lobectomy (6923 patients [70.1%]) and had a minimally invasive incision (4670 patients [53.3%]); however, adequate lymph node sampling (ie, ≥10 lymph nodes^25^) was performed among 3045 patients (31.0%). Histological results revealed that most patients had adenocarcinomas (5236 patients [52.9%]) with tumor grades greater than I (grade II, 4808 patients [52.5%]; grade III, 3022 patients [33.0%]; grade IV, 135 patients [1.5%]). At 30 days, 203 patients had died (2.1%) and 810 patients (8.2%) had been readmitted. Additional demographic and perioperative variables are shown in Table 1.

Pathologic upstaging occurred in 1171 patients (12.3%), and positive surgical margins were found in 309 patients (3.1%). Factors associated with higher odds of upstaging included younger age (odds ratio [OR] for every 1-year increase in age, 0.985; 95% CI, 0.975-0.996; *P* = .006), higher tumor grade (eg, III vs I; OR, 3.145; 95% CI, 2.341-4.224; *P* < .001), larger tumor size (eg, 31-40 mm vs <10 mm; OR, 1.734; 95% CI, 1.250-2.407; *P* = .03), greater number of lymph nodes examined (eg, ≥10 vs <10; OR, 1.476; 95% CI, 1.275-1.709; *P* = .009), and pneumonectomy (OR vs lobectomy, 3.528; 95% CI, 2.415-5.153; *P* < .001) (eTable 1 in the Supplement). Factors associated with higher odds of positive surgical margins included wedge resection (OR vs lobectomy, 2.501; 95% CI, 1.866-3.352; *P* < .001) and larger tumor size (eg, ≥40 mm vs <10 mm; OR, 3.087; 95% CI, 1.651-5.771; *P* < .001) (eTable 2 in the Supplement). Restricted cubic spline functions did not reveal a significant association between RTTS and the likelihood of upstaging or a resection with positive margins.

**Figure 2.**
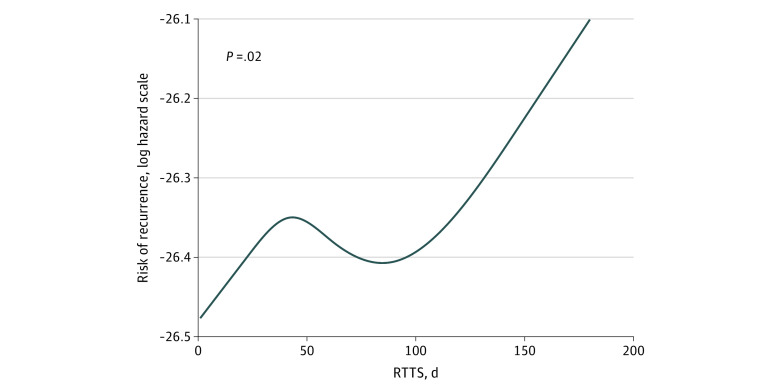
Association Between Radiological Time to Surgical Treatment (RTTS) and Probability of Recurrence Restricted cubic spline model presents the association between RTTS and the probability of recurrence.

With median (IQR) follow-up of 6.15 (2.51-11.51) years, recurrence was detected in 4158 patients (42.0%), with median (IQR) time to recurrence of 1.24 (0.43-2.76) years. Factors associated with increased risk of recurrence included younger age (hazard ratio [HR] for every 1 year increase in age, 0.992; 95% CI, 0.987-0.997; *P* = .002), higher Charlson Comorbidity Index score (HR for every 1 unit increase in composite score, 1.055; 95% CI, 1.037-1.073; *P* < .001), segmentectomy (HR vs lobectomy, 1.352; 95% CI, 1.179-1.551; *P* < .001) or wedge resection (HR vs lobectomy, 1.282; 95% CI, 1.179-1.394; *P* < .001), higher tumor grade (eg, II vs I; HR, 1.210; 95% CI, 1.085-1.349; *P* < .001), larger tumor size (eg, 31-40 mm vs <10 mm; HR, 1.209; 95% CI, 1.051-1.390; *P* = .008), lower number of lymph nodes examined (eg, ≥10 vs <10; HR, 0.866; 95% CI, 0.803-0.933; *P* < .001), higher pathologic stage (eg, III vs I, HR 1.571; 95% CI, 1.351-1.837; *P* < .001), and longer RTTS (eTable 3 in the Supplement). Based on the spline analysis, the risk of recurrence increased after approximately 12 weeks of delay (Figure 2). For each week of surgical delay beyond 12 weeks, the hazard for recurrence increased by 0.4% (HR, 1.004; 95% CI, 1.001-1.006; *P* = .002) (eFigure 1 in the Supplement).

**Figure 3.**
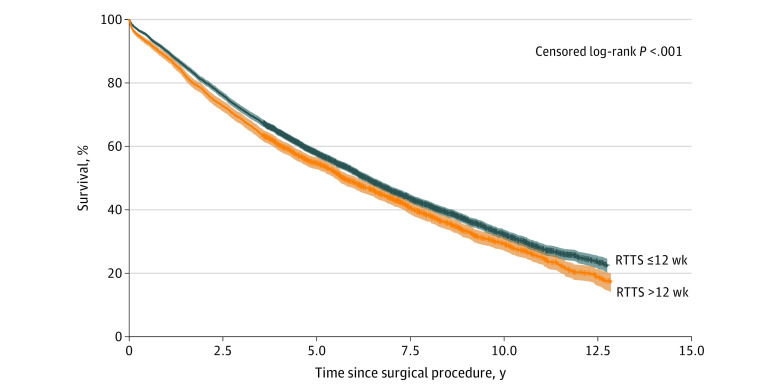
Overall Survival Following Delayed Surgical Treatment This Kaplan-Meier curve shows patients with clinical stage I non–small cell lung cancer with delayed (ie, >12 weeks radiological time to surgical treatment [RTTS]) vs nondelayed (≤12 weeks RTTS) surgical treatment.

To understand factors associated with surgical procedures delayed longer than 12 weeks, multivariable logistic regression analysis was performed (Table 2). Factors associated with delayed surgical treatment included African American race (OR vs White race, 1.267; 95% CI, 1.112-1.444; *P* < .001), higher ADI score (OR every 1 unit increase in ADI score, 1.005; 95% CI, 1.002-1.007; *P* = .002), lower hospital case load (OR for every 1 unit increase in case load, 0.998; 95% CI, 0.998-0.999; *P* = .001), year of diagnosis (ie, more recent cases less likely to have delay; OR for each additional year, 0.900; 95% CI, 0.884-0.915; *P* < .001), and performance of a preoperative mediastinoscopy or endobronchial ultrasonography (OR, 1.385; 95% CI, 1.226-1.564; *P* < .001). Smoking status, CCI score, and frailty score were not associated with longer RTTS. Patients receiving surgical treatment within 12 weeks of diagnosis had significantly better overall survival compared with patients who had treatment delayed more than 12 weeks (HR, 1.132; 95% CI, 1.064-1.204; *P* < .001) (Figure 3; eFigure 2 and eTable 4 in the Supplement).

**Table 2.** Factors Independently Associated With Delayed Surgical Treatment

Variable^a^	OR (95% CI)	*P* value
Age, y	1.005 (0.998-1.012)	.16
Female vs male sex	0.993 (0.768-1.284)	.96
Race		
White	[1 Reference]	NA
Black	1.267 (1.112-1.444)	<.001
Other^b^	1.361 (0.918-2.018)	.35
BMI	0.992 (0.983-1.001)	.09
Smoking status		
Current	[1 Reference]	NA
Former	1.031 (0.931-1.141)	.46
Never	1.307 (0.766-2.230)	.35
CCI score	0.980 (0.956-1.004)	.10
ADI score	1.005 (1.002-1.007)	.002
Surgical treatment year	0.900 (0.884-0.915)	<.001
Yearly hospital caseload^c^	0.998 (0.998-0.999)	.001
Endobronchial ultrasound or mediastinoscopy vs none	1.385 (1.226-1.564)	<.001
Histology		
Adenocarcinoma	[1 Reference]	NA
Squamous cell carcinoma	0.923 (0.829-1.028)	.78
Other	0.880 (0.751-1.033)	.27
Tumor grade		
I	[1 Reference]	NA
II	0.906 (0.781-1.051)	.94
III	0.883 (0.753-1.036)	.74
IV	0.827 (0.535-1.276)	.58
Tumor size, mm		
0-10	[1 Reference]	NA
11-20	1.132 (0.943-1.359)	.07
21-30	1.212 (1.003-1.465)	.004
31-40	0.991 (0.804-1.222)	.55
≥40	0.999 (0.782-1.277)	.71
Unknown	0.885 (0.585-1.339)	.35

Abbreviations: ADI, area deprivation index; CCI, Charlson Comorbidity Index; NA, not applicable; OR, odds ratio.

^a^ ORs for continuous variables are per 1-unit increase.

^b^ Other category includes any code other than White or Black in the American College of Surgery Facility Oncology Registry Data Standards manual.

^c^ Hospital caseload was measured as the number of patients with a lung cancer diagnosis at that institution in the year prior to a patient’s surgical procedure.

Finally, a sensitivity analysis was performed to assess whether CTTS was associated with recurrence (eFigure 3 in the Supplement). We found that CTTS was not associated with increased risk of recurrence when using this definition in the spline modeling.

## Discussion

This cohort study examined the association of delayed surgical treatment with oncologic outcomes in patients in the VHA with clinical stage I lung cancer. Using a more robust and precise method for quantifying surgical delay (ie, RTTS), our study found that patients who waited more than 12 weeks for resection had an increased risk of recurrence. There was no association between delayed surgical treatment and the likelihood of pathologic upstaging or resection with positive margins. These findings suggest that while patients with clinical stage I lung cancer should continue to undergo expedient resection, there may be only a modest biologic penalty associated with short-term delays (ie, those less than 3 months) if additional workup or optimization are required.

Our findings fill an important gap in the medical literature and overcome some of the limitations of previous publications, including those from our own group.^4,26^ Prior studies have provided conflicting information regarding the association of delayed surgical treatment with oncologic outcomes, with some finding an association^4,5,6,7,27^ and others finding no association.^8,9,10^ Most of these reports used arbitrary cutoffs to define delayed surgical treatment, a method that is inherently flawed.^4,5,6,8,9^ To address this shortcoming, we employed restricted cubic spline models, allowing us to examine the continuous association between TTS and the risk of each outcome. Based on the shape of these models, we found that surgical procedures delayed beyond 12 weeks had a significantly higher risk of recurrence. Furthermore, with access to detailed VHA data and by using validated methods,^21,28,29^ we were able to examine the risk of cancer recurrence, an outcome that is not available in most other large cancer databases, such as the NCDB. This is a more specific oncologic outcome than overall survival and is critical to consider in the context of treatment delays. Most prior publications about delayed surgical treatment have analyzed single-institution or NCDB data. Unfortunately, the date of diagnosis coded in the NCDB and related databases, including the VHA database, is fundamentally unreliable. Patients can be diagnosed by any combination of imaging results, symptoms, clinical judgement, pathology, and cytology, and this diagnosis can even be changed in retrospect by the coders.^15^ As Figure 1 shows, according to this coding algorithm, close to 30% of VHA patients were “diagnosed” with cancer on the same day as their operation; importantly, this is an observation that is also seen in the NCDB.^7^ This is clearly problematic given that each of these patients indeed had cancer with some indicator of diagnosis (likely imaging) at an unknown interval before the date of surgical treatment. Therefore, analyzing this variable in these databases, even after excluding those patients with no documented wait time, is potentially flawed.

It is worth noting that veterans appear to wait longer for surgical treatment than the general population. According to the CTTS definition (even though it is flawed), patients waited a mean of 48.5 days (7 weeks) between diagnosis and surgical treatment. Yang and colleagues^7^ performed an analysis of patients in the NCDB with clinical stage Ia squamous cell carcinoma who were undergoing lobectomy. The median wait time to surgical treatment was 38 days (5.5 weeks). While this discrepancy warrants further study, veterans appear to have a high comorbidity burden while maintaining similar rates of short-term complications as the general population.^4,7^ It is possible that the observed delays among veterans allow for more complex care of patients with more severe illness. If comorbidities are the driving factor associated with delays, then marginally delayed surgical procedures seem acceptable and likely necessary.

Timely surgical treatment has been proposed as a quality metric for lung cancer care.^30,31^ Our study provides evidence that patients with clinical stage I disease should ideally receive resection within 12 weeks from the date of CT imaging that suggests lung cancer. However, there are several caveats to including a 12-week period as a proposed quality measure. Timely surgical treatment depends on several disease-specific and patient-specific factors.^32,33^ For instance, patients requesting second opinions often experience delays in care. Additionally, as our study noted, several socioeconomic factors are associated with timeliness of care. Many of these variables remain nonmodifiable in the short interval between lung cancer diagnosis and surgical treatment, and treating institutions should not incur a penalty for factors outside their control.^34,35^ Nonetheless, long-term, targeted interventions may improve access to care and eliminate disparities in timely care between different subpopulations of patients with lung cancer.

Our study has some important strengths. First, we have assembled a relatively uniform population of patients with lung cancer, with veterans having access to universal health care coverage under the VHA. This eliminates confounding due to insurance-related factors previously noted to be associated with timely care.^13^ Second, with access to data on cancer recurrence and the use of restricted cubic spline analysis, we were able to use appropriate statistical methodology to assess long-term oncologic outcomes. Finally, our data provide particularly timely information regarding delayed medical care, a common issue during the ongoing COVID-19 pandemic.

### Limitations

Our study also has some limitations. First, our method for quantifying surgical delay based on the date of CT imaging may be imperfect. Because lung cancers have heterogeneous CT findings, our study results do not provide information on how quickly to proceed when dealing with indeterminate imaging findings. Second, our study assesses pathologically confirmed cases of NSCLC, but definitive pathologic confirmation is often unavailable preoperatively, especially for clinical stage I disease. The decision to proceed to surgical treatment as opposed to continued surveillance can be complex, especially with smaller lung nodules, which can challenge the utility of time to treatment standards in real-world practice. Our data rather suggest that for highly suspicious nodules consistent with clinic stage I disease (and certainly those with preoperative confirmatory pathology), surgical treatment within at least 12 weeks of radiographic diagnosis is prudent. Third, our study consisted exclusively of a veteran population. While this cohort is not uniformly comparable to the overall US population, the general patterns of lung cancer care and outcomes are similar between veterans and nonveterans.^36^ Hence, our findings are likely to be relevant for the broad population of patients with early-stage lung cancer.

## Conclusions

These findings suggest that veterans with clinical stage I lung cancer who wait more than 12 weeks for resection may have an increased risk of recurrence and worse survival rates. Efforts to minimize delays in surgical procedures for lung cancer are essential to decrease the risk of disease recurrence and the associated worse prognosis. Such endeavors are particularly important in the face of compromised access to care during the ongoing COVID-19 pandemic, but also instrumental in ensuring timely care at a programmatic level for a disease that is the leading cause of cancer-related mortality in the US.

## Notes

Supplement. **eAppendix.** Supplemental Methods **eFigure 1.** Cumulative Incidence Function for Recurrence **eFigure 2.** Direct Adjusted Survival Curve Following Delayed Surgical Treatment **eFigure 3.** Association Between Clinical Time to Surgical Treatment and Probability of Recurrence **eTable 1.** Multivariable Analysis of Factors Associated With Upstaging **eTable 2.** Multivariable Analysis of Factors Associated With Positive Surgical Margin **eTable 3.** Multivariable Analysis of Factors Associated With Cancer Recurrence **eTable 4.** Multivariable Analysis of Factors Associated With Overall Survival
