# Supplementary material for: Analysis of Delayed Surgical Treatment and Oncologic Outcomes in Clinical Stage I Non–Small Cell Lung Cancer
Source: JAMA Netw Open. 2021 May 27;4(5):e2111613. doi: 10.1001/jamanetworkopen.2021.11613 (PMC8160592; doi:10.1001/jamanetworkopen.2021.11613)
Supplement: Supplement. — eAppendix. Supplemental Methods eFigure 1. Cumulative Incidence Function for Recurrence eFigure 2. Direct Adjusted Survival Curve Following Delayed Surgical Treatment eFigure 3. Association Between Clinical Time to Surgical Treatment and Probability of Recurrence eTable 1. Multivariable Analysis of Factors Associated With Upstaging eTable 2. Multivariable Analysis of Factors Associated With Positive Surgical Margin eTable 3. Multivariable Analysis of Factors Associated With Cancer Recurrence eTable 4. Multivariable Analysis of Factors Associated With Overall Survival [file jamanetwopen-e2111613-s001.pdf]

Supplemental Online Content

Heiden BT, Eaton DB Jr, Engelhardt KE, et al. Analysis of delayed surgical treatment and oncologic outcomes in clinical stage I non–small cell lung cancer. *JAMA Netw Open*. 2021;4(5):e2111613. doi:10.1001/jamanetworkopen.2021.11613

eAppendix. Supplemental Methods

eFigure 1. Cumulative Incidence Function for Recurrence

eFigure 2. Direct Adjusted Survival Curve Following Delayed Surgical Treatment

eFigure 3. Association Between Clinical Time to Surgical Treatment and Probability of Recurrence

eTable 1. Multivariable Analysis of Factors Associated With Upstaging

eTable 2. Multivariable Analysis of Factors Associated With Positive Surgical Margin

eTable 3. Multivariable Analysis of Factors Associated With Cancer Recurrence

eTable 4. Multivariable Analysis of Factors Associated With Overall Survival

This supplemental material has been provided by the authors to give readers additional information about their work.

© 2021 Heiden BT et al. *JAMA Network Open.*

eAppendix. Supplementary Methods

Spline knots and locations.

Each spline model was constructed using 5 knots with the following locations:

**Knots for Spline Models**	
**Knot Number**	**TTS (days)**
1	23.0
2	42.0
3	62.0
4	88.0
5	148.0

Testing proportional hazards assumption for time variables.

ZPH tests for delay variable (>12 weeks) and spline terms in both cox proportional hazards model and recurrence competing risk model. (Note: a p-value of less than 0.05 indicates a violation of the PH assumption.)

**ZPH Test for Nonproportional Hazards (Overall Survival)**	
Time variable	P-value
Delayed surgery (>12 weeks)	0.2639
Spline term (#1)	0.3559
Spline term (#2)	0.3981
Spline term (#3)	0.4703
Spline term (#4)	0.6400

© 2021 Heiden BT et al. *JAMA Network Open.*

ZPH Test for Nonproportional Hazards (Recurrence)	
Time variable	P-value
Delayed surgery (>12 weeks)	0.4551
Spline term (#1)	0.1345
Spline term (#2)	0.2480
Spline term (#3)	0.2514
Spline term (#4)	0.2482

© 2021 Heiden BT et al. *JAMA Network Open.*

eFigure1. Cumulative Incidence Function for Recurrence.

Cumulative incidence function for recurrence using multivariable competing risk model based on several rTTS cutoffs (4-, 8-, 12-, 16-weeks).

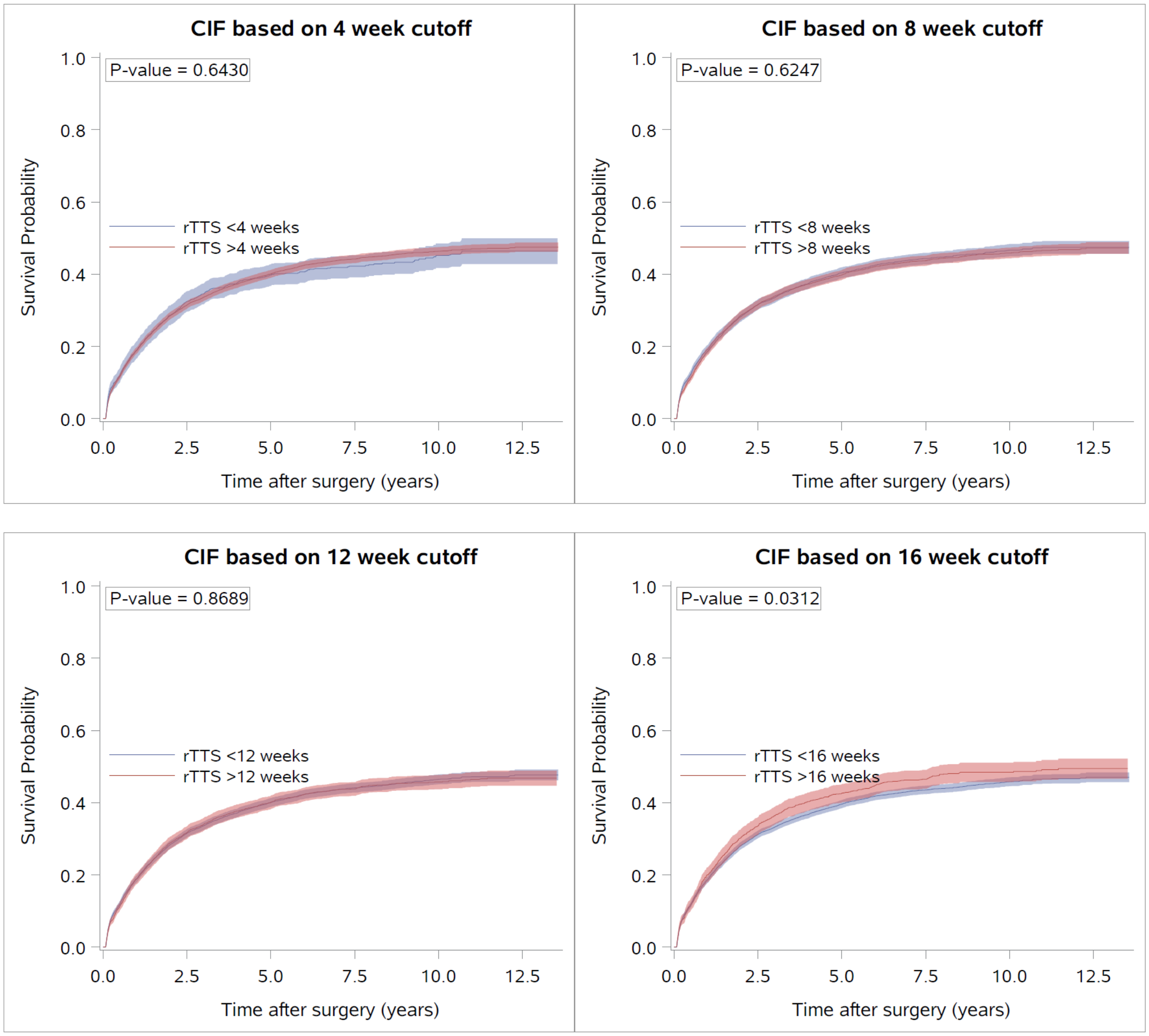

© 2021 Heiden BT et al. *JAMA Network Open.*

eFigure2. Direct Adjusted Survival Curve Following Delayed Surgical Treatment

Multivariable direct adjusted overall survival curve for clinical stage I NSCLC patients with delayed (>12 weeks) versus non-delayed (<12 weeks) surgery.

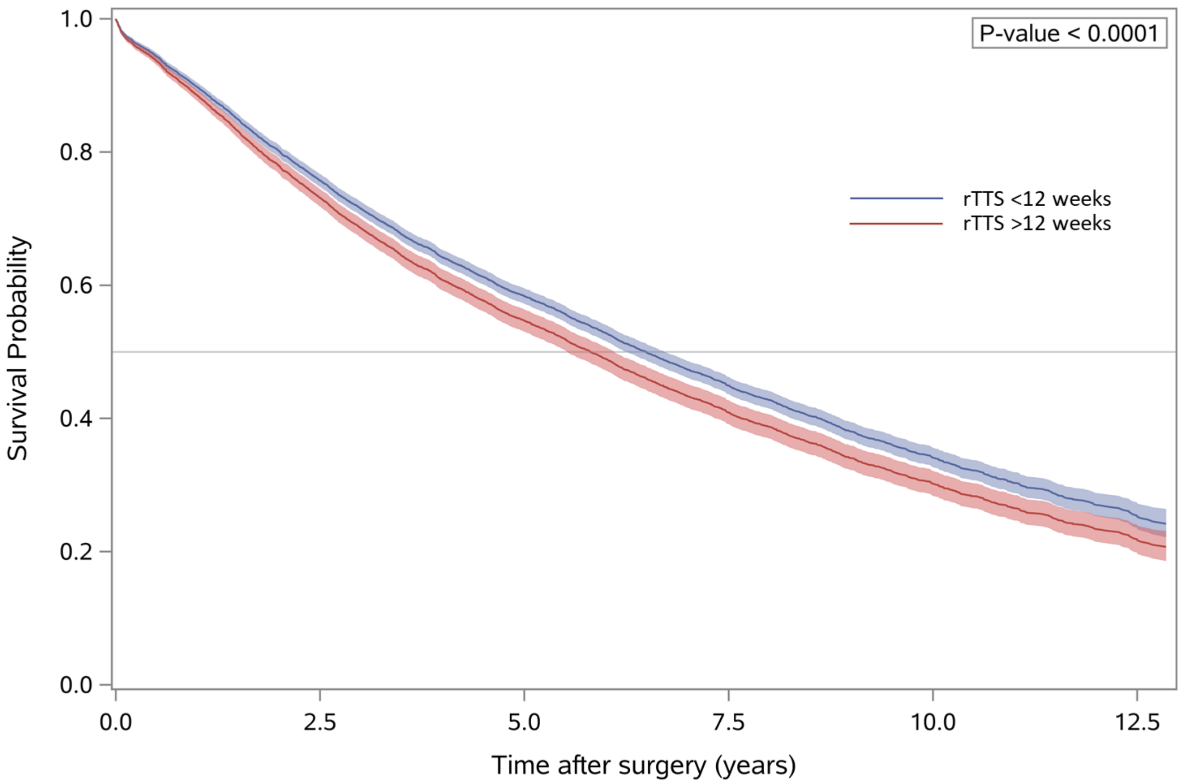

© 2021 Heiden BT et al. *JAMA Network Open.*

eFigure3. Association between Clinical Time to Surgical Treatment and Probability of Recurrence

Restricted cubic spline model for relationship between cTTS and recurrence.

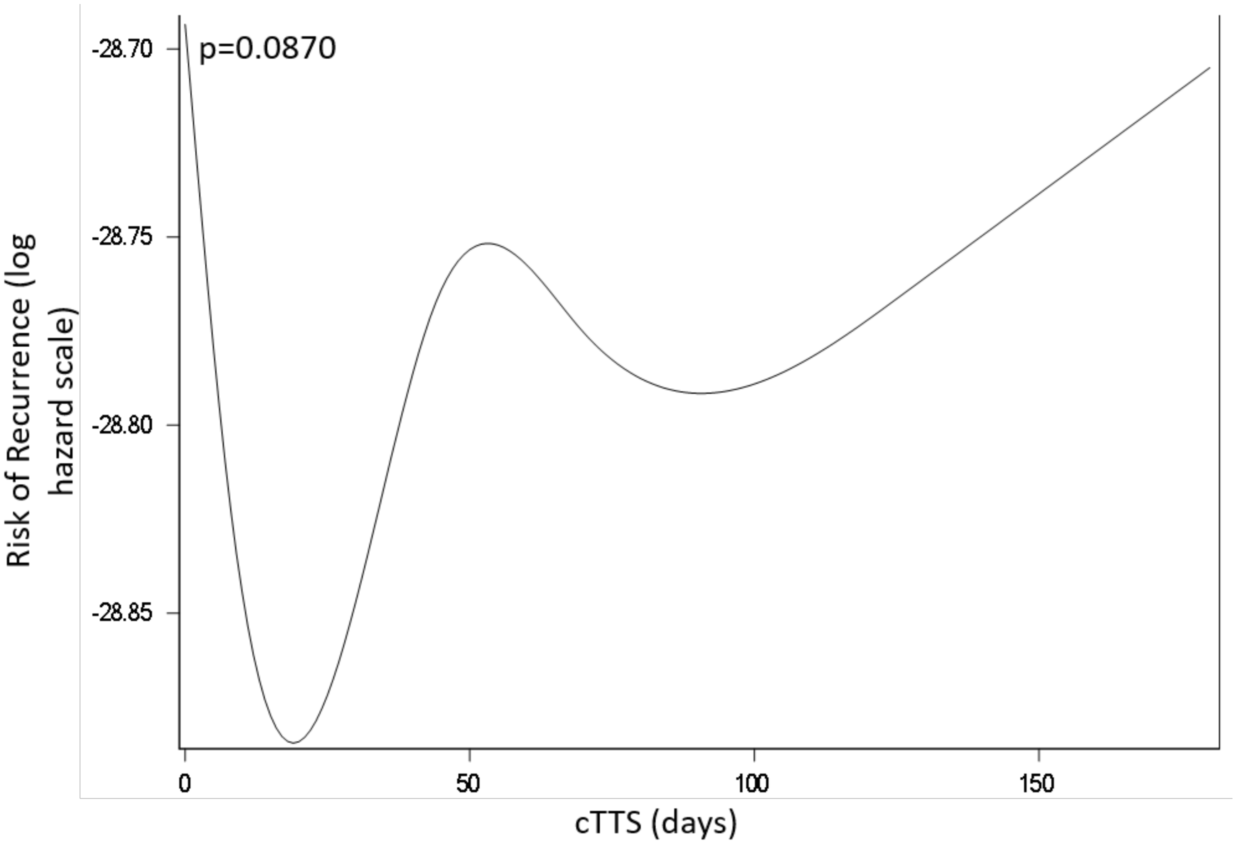

© 2021 Heiden BT et al. *JAMA Network Open.*

**eTable 1.** Multivariable Analysis of Factors Associated With Upstaging				
**Variable**	**Odds Ratio**	**95% Wald Confidence Limits**		**P-value**
**Age, y**	0.985	0.975	0.996	0.0061
**Female vs male, sex**	1.022	0.715	1.461	0.9062
**Race (reference = white)**				
**Black**	1.069	0.886	1.289	0.1739
**Other**	0.678	0.347	1.323	0.2178
**BMI**	1.022	1.009	1.035	0.0011
**Smoking status (reference = current)**				
**Former**	1.028	0.889	1.188	0.8602
**Never**	0.980	0.436	2.203	0.9342
**CCI**	1.003	0.968	1.039	0.8876
**ADI**	1.006	1.001	1.011	0.0161
**Surgical year, y**	1.040	1.014	1.067	0.0027
**Yearly hospital caseload, y**	1.001	1.000	1.002	0.1459
**EBUS or mediastinoscopy vs none**	1.148	0.971	1.357	0.1066
**Type of operation (reference = lobectomy)**				
**Pneumonectomy**	3.528	2.415	5.153	<.0001
**Segmentectomy**	0.620	0.431	0.892	<.0001
**Wedge**	0.826	0.678	1.007	0.0004
**Incision type (reference = thoracotomy)**				
**VATS**	0.878	0.754	1.022	0.0010
**Unknown**	1.318	1.045	1.663	0.0024
**Histology (reference = adenocarcinoma)**				
**Squamous cell carcinoma**	0.721	0.618	0.841	0.0073
**Other**	0.816	0.652	1.021	0.7168
**Grade (reference = I)**				
**II**	2.447	1.859	3.302	0.0907
**III**	3.145	2.341	4.224	<.0001
**IV**	2.638	1.405	4.954	0.3304
**Tumor size (reference = 0-10mm)**				
**11-20mm**	1.076	0.791	1.464	<.0001
**21-30mm**	1.645	1.206	2.242	0.0689
**31-40mm**	1.734	1.250	2.407	0.0273
**40+ mm**	2.545	1.789	3.619	<.0001
**Unknown**	1.138	0.662	1.957	0.2395
**Lymph node collection (reference <10)**				
**≥10**	1.476	1.275	1.709	0.0091
**Unknown**	1.352	1.017	1.797	0.4481

ADI, area deprivation index; CCI, Charlson comorbidity index; EBUS, endobronchial ultrasound; VATS, video-assisted thoracoscopic surgery

© 2021 Heiden BT et al. *JAMA Network Open.*

**eTable 2.** Multivariable Analysis of Factors Associated With Positive Surgical Margin				
**Variable**	**Odds Ratio**	**95% Wald Confidence Limits**		**P-value**
**Age, y**	0.983	0.965	1.002	0.0763
**Female vs male, sex**	1.410	0.767	2.592	0.2691
**Race (reference = white)**				
**Black**	1.057	0.752	1.487	0.7974
**Other**	0.940	0.293	3.010	0.8802
**BMI**	0.993	0.970	1.017	0.5675
**Smoking status (reference = current)**				
**Former**	1.166	0.892	1.524	0.9653
**Never**	<0.001	<0.001	>999.999	0.9660
**CCI**	1.053	0.990	1.120	0.1015
**ADI**	1.003	0.995	1.010	0.5055
**Surgical year, y**	0.994	0.948	1.042	0.7988
**EBUS or mediastinoscopy vs none**	0.920	0.655	1.292	0.6300
**Type of operation (reference = lobectomy)**				
**Pneumonectomy**	0.864	0.310	2.409	0.3355
**Segmentectomy**	1.197	0.668	2.144	0.8173
**Wedge**	2.501	1.866	3.352	0.0001
**Incision type (reference = thoracotomy)**				
**VATS**	0.915	0.690	1.212	0.3935
**Unknown**	1.081	0.696	1.679	0.5643
**Histology (reference = adenocarcinoma)**				
**Squamous cell carcinoma**	1.096	0.830	1.447	0.3712
**Other**	0.915	0.594	1.408	0.5219
**Grade (reference = I)**				
**II**	1.217	0.771	1.922	0.2830
**III**	1.883	1.183	2.996	0.0712
**IV**	1.852	0.656	5.233	0.4877
**Tumor size (reference = 0-10mm)**				
**11-20mm**	1.259	0.740	2.144	0.9607
**21-30mm**	1.703	0.988	2.935	0.1513
**31-40mm**	1.848	1.026	3.329	0.0915
**40+ mm**	3.087	1.651	5.771	0.0002
**Unknown**	0.346	0.045	2.647	0.1222
**Lymph node collection (reference <10)**				
**≥10**	0.822	0.612	1.104	0.9365
**Unknown**	0.698	0.373	1.305	0.4097

ADI, area deprivation index; CCI, Charlson comorbidity index; EBUS, endobronchial ultrasound;

VATS, video-assisted thoracoscopic surgery

© 2021 Heiden BT et al. *JAMA Network Open.*

**eTable 3.** Multivariable Analysis of Factors Associated With Cancer Recurrence				
**Variable**	**Hazard Ratio**	**95% Wald Confidence Limits**		**P-value**
**Age, y**	0.992	0.987	0.997	0.0025
**Female vs male, sex**	0.844	0.703	1.013	0.0687
**Race (reference = white)**				
**Black**	0.985	0.900	1.078	0.7451
**Other**	1.193	0.910	1.563	0.2006
**BMI**	0.994	0.988	1.001	0.0925
**Smoking status (reference = current)**				
**Former**	0.968	0.902	1.038	0.3582
**Never**	0.977	0.662	1.443	0.9087
**CCI**	1.055	1.037	1.073	<.0001
**ADI**	0.999	0.997	1.001	0.2680
**Surgical year, y**	0.986	0.974	0.999	0.0291
**Yearly hospital caseload, y**	1.000	1.000	1.001	0.3970
**EBUS or mediastinoscopy vs none**	1.063	0.976	1.158	0.1614
**Type of operation (reference = lobectomy)**				
**Pneumonectomy**	0.832	0.620	1.117	0.2210
**Segmentectomy**	1.352	1.179	1.551	<.0001
**Wedge**	1.282	1.179	1.394	<.0001
**Incision type (reference = thoracotomy)**				
**VATS**	0.943	0.876	1.014	0.1126
**Unknown**	0.860	0.755	0.981	0.0243
**Histology (reference = adenocarcinoma)**				
**Squamous cell carcinoma**	0.957	0.887	1.031	0.2477
**Other**	1.143	1.029	1.270	0.0131
**Grade (reference = I)**				
**II**	1.210	1.085	1.349	0.0006
**III**	1.348	1.201	1.513	<.0001
**IV**	1.438	1.089	1.899	0.0105
**Tumor size (reference = 0-10mm)**				
**11-20mm**	0.911	0.806	1.029	0.1326
**21-30mm**	1.001	0.881	1.137	0.9922
**31-40mm**	1.209	1.051	1.390	0.0079
**40+ mm**	1.379	1.169	1.627	0.0001
**Unknown**	1.249	0.980	1.592	0.0722
**Lymph node collection (reference <10)**				
**≥10**	0.866	0.803	0.933	0.0002
**Unknown**	0.958	0.832	1.103	0.5519
**30-day readmission vs none**	1.125	0.996	1.270	0.0576
**Pathologic stage (reference = I)**				

© 2021 Heiden BT et al. *JAMA Network Open.*

**II**	1.121	0.992	1.266	0.0675
**III**	1.575	1.351	1.837	<.0001

ADI, area deprivation index; CCI, Charlson comorbidity index; EBUS, endobronchial ultrasound;

VATS, video-assisted thoracoscopic surgery

© 2021 Heiden BT et al. *JAMA Network Open.*

**eTable4.** Multivariable Analysis of Factors Associated With Overall Survival				
**Variable**	**Hazard Ratio**	**95% Wald Confidence Limits**		**P-value**
**rTTS (reference <12 weeks)**	1.132	1.064	1.204	<.0001
**Age, y**	1.022	1.017	1.026	<.0001
**Female vs male, sex**	0.721	0.599	0.868	0.0005
**Race (reference = white)**				
**Black**	0.816	0.750	0.887	<.0001
**Other**	0.821	0.629	1.073	0.1487
**BMI**	0.976	0.970	0.981	<.0001
**Smoking status (reference = current)**				
**Former**	0.821	0.772	0.873	<.0001
**Never**	0.554	0.373	0.823	0.0034
**CCI**	1.112	1.096	1.128	<.0001
**ADI**	1.001	0.999	1.002	0.6034
**Surgical year, y**	0.972	0.961	0.983	<.0001
**Yearly hospital caseload, y**	0.999	0.999	1.000	0.0142
**EBUS or mediastinoscopy vs none**	1.068	0.990	1.151	0.0874
**Type of operation (reference = lobectomy)**				
**Pneumonectomy**	1.244	1.009	1.534	0.0408
**Segmentectomy**	1.073	0.942	1.222	0.2879
**Wedge**	1.289	1.198	1.386	<.0001
**Incision type (reference = thoracotomy)**				
**VATS**	1.024	0.961	1.091	0.4691
**Unknown**	0.992	0.889	1.108	0.8914
**Histology (reference = adenocarcinoma)**				
**Squamous cell carcinoma**	1.143	1.072	1.219	<.0001
**Other**	1.147	1.045	1.260	0.0039
**Grade (reference = I)**				
**II**	1.196	1.083	1.322	0.0004
**III**	1.355	1.220	1.504	<.0001
**IV**	1.368	1.066	1.756	0.0138
**Tumor size (reference = 0-10mm)**				
**11-20mm**	1.020	0.913	1.139	0.7289
**21-30mm**	1.073	0.957	1.203	0.2292
**31-40mm**	1.254	1.107	1.421	0.0004
**40+ mm**	1.256	1.087	1.452	0.0020
**Unknown**	0.569	0.418	0.774	0.0003
**Lymph node collection (reference <10)**				
**≥10**	0.924	0.865	0.987	0.0182
**Unknown**	0.999	0.884	1.128	0.9855
**30-day readmission vs none**	1.153	1.040	1.277	0.0066

© 2021 Heiden BT et al. *JAMA Network Open.*

**Pathologic stage (reference = I)**				
**II**	1.389	1.252	1.541	<.0001
**III**	1.968	1.728	2.241	<.0001
**Recurrence (reference = none)**	1.391	1.313	1.474	<.0001

ADI, area deprivation index; CCI, Charlson comorbidity index; EBUS, endobronchial ultrasound;

VATS, video-assisted thoracoscopic surgery

© 2021 Heiden BT et al. *JAMA Network Open.*
